# Dopamine and cAMP-regulated phosphoprotein 32 kDa (DARPP-32) and survival in breast cancer: a retrospective analysis of protein and mRNA expression

**DOI:** 10.1038/s41598-019-53529-z

**Published:** 2019-11-18

**Authors:** Shreeya Kotecha, Marie N. Lebot, Bhudsaban Sukkarn, Graham Ball, Paul M. Moseley, Stephen Y. Chan, Andrew R. Green, Emad Rakha, Ian O. Ellis, Stewart G. Martin, Sarah J. Storr

**Affiliations:** 1 Nottingham Breast Cancer Research Centre, Division of Cancer and Stem Cells, School of Medicine, University of Nottingham, Nottingham City Hospital, Nottingham, NG5 1PB UK; 2 John van Geest Cancer Research Centre, School of Science and Technology, Nottingham Trent University, Clifton Campus, Nottingham, NG1 4BU UK

**Keywords:** Breast cancer, Prognostic markers, Breast cancer, Tumour biomarkers

## Abstract

Dopamine and cAMP regulated phosphoprotein 32 kDa (DARPP-32) also known as phosphoprotein phosphatase-1 regulatory subunit 1B and encoded by the PPP1R1B gene is an inhibitor of protein phosphatase-1 and protein kinase A. DARPP-32 is expressed in a wide range of epithelial cells and some solid tumours; however, its role in breast cancer is only partially defined. DARPP-32 expression was determined using immunohistochemistry in two independent cohorts of early stage invasive breast cancer patients (discovery n = 1352; validation n = 1655), and 112 HER2 positive breast cancer patients treated with trastuzumab and adjuvant chemotherapy. *PPP1R1B* mRNA expression was assessed in the METABRIC cohort (n = 1980), using artificial neural network analysis to identify associated genes. In the discovery cohort, low nuclear expression of DARPP-32 was significantly associated with shorter survival (*P* = 0.041), which was independent of other prognostic variables (*P* = 0.019). In the validation cohort, low cytoplasmic and nuclear expression was significantly associated with shorter survival (both *P* = 0.002), with cytoplasmic expression independent of other prognostic variables (*P* = 0.023). Stronger associations with survival in oestrogen receptor (ER) positive disease were observed. In patients treated with trastuzumab, low nuclear expression was significantly associated with adverse progression-free survival (*P* = 0.031). In the METABRIC cohort, low *PPP1R1B* expression was associated with shortened survival of ER positive patients. Expression of *CDC42* and *GRB7*, amongst others, were associated with *PPP1R1B* expression. This data suggests a role for DARPP-32 as a prognostic marker with clinical utility in breast cancer.

## Introduction

Dopamine and cAMP regulated phosphoprotein 32 kDa (DARPP-32) also known as phosphoprotein phosphatase-1 regulatory subunit 1B and encoded by the *PPP1R1B* gene, was first described in 1983^1^ and has been widely characterised as a signalling protein highly concentrated in brain regions rich in dopaminergic nerve terminals^2–4^. DARPP-32 was originally demonstrated to be a potent inhibitor of protein phosphatase-1 (PP-1) and a substrate of calcineurin^5,6^. Protein kinase A (PKA) phosphorylation of Thr34 allows DARPP-32 to inhibit protein phosphatase-1 (PP-1); cyclin dependent kinase (CDK)-5 phosphorylation of Thr75 allows DARPP-32 to inhibit PKA and enhance β-adducin Ser713 phosphorylation^7^. A truncated DARPP-32 isoform, t-DARPP, lacks the Thr-34 phosphorylation site and was originally identified in gastric cancer^8^. Interaction between DARPP-32, calcineurin and Bcl-2 assists with the anti-apoptotic function of Bcl-2 by preventing Ca^2+^ induced cell death through interaction with inositol 1,4,5-triphosphate receptor (InsP_3_R)^9^.

DARPP-32 activation is regulated by an array of neurotransmitters, such as dopamine, glutamate, serotonin and adenosine, but has also been shown to mediate the actions of multiple drugs of abuse, including cocaine, amphetamine, nicotine and caffeine (reviewed in^10^). DARPP-32 has been implicated in a number of psychiatric and neurological disorders, such as schizophrenia. In addition to the central nervous system, DARPP-32 is expressed in a wide range of epithelial cells^11^.

High levels of DARPP-32 in colorectal cancer are associated with survival of Dukes A and B patients^12^, and in glioblastoma, high DARPP-32/STAT3 and DARPP-32/STAT5B mRNA ratios are associated with longer progression free survival and overall survival^13^. In gastric cancer, DARPP-32 can promote cell invasion through CXCR4-mediated activation of the MT1-MMP/MMP-2 pathway^14^. A PPP1R1B-STARD3 fusion transcript has also been identified in gastric cancer, that increases *in vitro* cell proliferation through the phosphatidylinositol-3-kinase (PI3K)/AKT pathway^15^. DARPP-32 has been shown to influence breast epithelial cell migration; in MCF-7 and MDA-MB-231 cells this has been shown to be in a DDR1 dependent manner^16^. DARPP-32 phosphorylation, induced by Wnt-5a, has also been shown to inhibit MCF-7 cell migration in a CREB-dependent manner^17^.

The truncated splice variant, t-DARPP is present in gastric, breast, prostate, colon and stomach cancers^8,11^, and in models of murine tumourigenesis, DARPP-32 expression is expressed in normal mouse tissue and some breast tumours, with t-DARPP expressed only within tumours^18^. In breast cancer, t-DARPP mRNA is expressed in 36% of primary breast cancers (n = 36) relative to adjacent normal breast tissues (n = 18)^19^. Interestingly, the expression of t-DARPP has been implicated in resistance to the HER2 targeted agent, trastuzumab, in HER2 positive breast cancer cells via sustained signalling through phosphatidylinositol-4,5 bisphosphate 3-kinase (PI3K)/akt pathway and activation of PKA *in vitro*^19–23^, and in conferring a survival advantage to the HER2 targeted agent, lapatinib^24^. The role of DARPP-32 and t-DARPP in cancer is reviewed in^25^.

We sought to determine the frequency and importance of DARPP-32 expression in two large independent cohorts of early stage invasive breast cancer patients, including an additional cohort of HER2 positive breast cancer patients treated with trastuzumab to examine if DARPP-32 was associated with patient survival. In addition to protein expression, we sought to assess *PPP1R1B* mRNA expression in a large, well-annotated series of breast cancer patients, including artificial neural network analysis to identify genes associated with *PPP1R1B* expression.

## Methods

### Patient cohorts

This study is reported according to reporting recommendations for tumour marker prognostic studies (REMARK) criteria^26^. For protein expression three well-characterised patient cohorts were used; the discovery cohort, functioned as a discovery set; the validation cohort, functioned as a validation set and the HER2 cohort was used to assess DARPP-32 expression in HER2 positive patients treated with trastuzumab. Breast cancer specific survival was calculated as the time interval between primary surgery and death resultant from breast cancer. Progression-free survival was defined as the date of surgery to relapse (including local and regional relapse).

### Discovery cohort

1352 early stage invasive breast cancer patients were available for assessment in the discovery cohort, with all patients treated at Nottingham University Hospitals between 1987 and 1998. All patients were managed in a standard manner, where all patients underwent a mastectomy or wide local excision, as decided by disease characteristics or patient choice, followed by radiotherapy if indicated. Patients received systemic adjuvant treatment on the basis of Nottingham Prognostic index (NPI), oestrogen receptor (ER), and menopausal status. Patients with an NPI score less than 3.4 did not receive adjuvant treatment and patients with an NPI score of 3.4 and above were candidates for CMF combination chemotherapy (cyclophosphamide, methotrexate and 5-fluorouracil) if they were ER negative or premenopausal; and hormonal therapy if they were ER positive. Median follow-up was 205 months determined using the reverse Kaplan-Meier method and clinicopathological information for this cohort is available in Table 1.

**Table 1:** Associations between the cytoplasmic and nuclear expression of DARPP-32, determined in the discovery cohort and validation cohort using immunohistochemistry, with clinicopathological variables.

	Discovery cohort						Validation cohort					
	Cytoplasmic expression			Nuclear expression			Cytoplasmic expression			Nuclear expression		
	Low	High	*P* value	Low	High	*P* value	Low	High	*P* value	Low	High	*P* value
**Age**												
<50 years	221 (16.4%)	238 (17.6%)	0.470	295 (21.8%)	164 (12.1%)	0.467	222 (13.5%)	283 (17.3%)	0.881	272 (16.6%)	233 (14.2%)	0.251
≥50 years	448 (33.2%)	444 (32.9%)		591 (43.7%)	301 (22.3%)		494 (30.1%)	640 (39.0%)		576 (35.1%)	558 (34.0%)	
**Tumour size**												
<20 mm	414 (30.8%)	397 (29.5%)	0.188	594 (40.8%)	262 (19.5%)	0.052	422 (25.8%)	589 (36.0%)	**0.048**	513 (31.3%)	334 (20.4%)	0.320
³20 mm	253 (18.8%)	281 (20.9%)		334 (24.8%)	200 (14.9%)		293 (17.9%)	334 (20.4%)		498 (30.4%)	293 (17.9%)	
**T stage**												
1	404 (30.0%)	415 (30.9)	0.210	536 (39.9%)	283 (21.0%)	0.500	424 (25.9%)	608 (37.2%)	**0.016**	501 (30.6%)	531 (32.5%)	**0.001**
2	215 (16.0%)	197 (14.6%)		277 (20.6%)	135 (10.0%)		208 (12.7%)	237 (14.5%)		244 (14.9%)	201 (12.3%)	
3	49 (3.6%)	65 (4.8%)		70 (5.2%)	44 (3.3%)		81 (5.0%)	77 (4.7%)		100 (6.1%)	58 (3.5%)	
**Tumour grade**												
1	104 (7.7%)	124 (9.2%)	0.165	153 (11.4%)	75 (5.6%)	0.391	84 (5.1%)	179 (10.9%)	<**0.001**	105 (6.4%)	158 (9.6%)	<**0.001**
2	238 (17.7%)	212 (15.8%)		304 (22.6%)	146 (10.9%)		300 (18.3%)	358 (21.9%)		349 (21.3%)	309 (18.9%)	
3	325 (24.2%)	342 (25.4%)		426 (31.7%)	241 (17.9%)		331 (20.2%)	386 (23.6%)		393 (24.0%)	324 (19.8%)	
**ER status**												
Negative	132 (10.1%)	216 (16.5%)	<**0.001**	173 (13.2%)	175 (13.4%)	<**0.001**	90 (5.5%)	230 (14.0%)	<**0.001**	114 (7.0%)	206 (12.6%)	<**0.001**
Positive	518 (39.5%)	444 (33.9%)		683 (52.1%)	279 (21.3%)		626 (38.2%)	694 (42.3%)		734 (44.8%)	586 (35.7%)	
**PgR status**												
Negative	247 (19.4%)	301 (23.7%)	**0.007**	319 (25.1%)	229 (18.0%)	<**0.001**	246 (15.9%)	405 (26.1%)	<**0.001**	299 (19.3%)	352 (22.7%)	<**0.001**
Positive	381 (30.0%)	341 (26.9%)		513 (40.4%)	249 (16.5%)		425 (27.4%)	473 (30.5%)		502 (32.4%)	396 (25.6%)	
**NPI category**												
Good (≤3.4)	199 (14.8%)	208 (15.5%)	0.810	275 (20.5%)	132 (9.8%)	0.432	230 (14.1%)	339 (20.7%)	**0.002**	275 (16.8%)	294 (18.0%)	<**0.001**
Intermediate (3.41–5.4)	348 (25.9%)	342 (25.5%)		452 (33.7%)	238 (17.7%)		347(21.2%)	465 (28.5%)		405 (24.8%)	407 (24.9%)	
Poor (>5.4)	119 (8.9%)	127 (9.5%)		154 (11.5%)	92 (6.9%)		135 (8.3%)	118 (7.2%)		164 (10.0%)	89 (5.4%)	
**Tubule formation**												
1	31 (2.4%)	44 (3.4%)	0.218	43 (3.3%)	32 (2.5%)	0.160	28 (1.7%)	92 (5.7%)	<**0.001**	43 (2.6%)	77 (4.7%)	<**0.001**
2	201 (15.5%)	219 (16.9%)		286 (22.1%)	134 (10.3%)		184 (11.3%)	295 (18.2%)		219 (13.5%)	260 (16.0%)	
3	408 (31.5%)	394 (30.4%)		519 (40.0%)	283 (21.8%)		494 (30.4%)	531 (32.7%)		573 (35.3%)	452 (27.8%)	
**Pleomorphism**												
1	9 (0.7%)	17 (1.3%)	0.179	14 (1.1%)	12 (0.9%)	**0.031**	5 (0.3%)	19 (1.2%)	**0.016**	10 (0.6%)	14 (0.9%)	0.081
2	267 (20.6%)	252 (19.5%)		360 (27.8%)	159 (12.3%)		200 (12.3%)	294 (18.1%)		236 (14.5%)	258 (15.9%)	
3	36 (28.0%)	387 (29.9)		473 (36.5%)	277 (21.4%)		501 (30.8%)	605 (37.3%)		589 (36.3%)	517 (31.8%)	
**Mitosis**												
1	213 (16.4%)	239 (18.4%)	0.093	293 (22.6%)	159 (12.3%)	0.241	336 (20.7%)	469 (28.9%)	**0.034**	392 (24.2%)	413 (25.5%)	**0.007**
2	138 (10.6%)	111 (8.6%)		174 (13.4%)	75 (5.8%)		153 (9.4%)	152 (9.4%)		181 (11.2%)	124 (7.6%)	
3	289 (22.3%)	307 (23.7%)		381 (29.4%)	215 (16.6%)		217 (13.4%)	295 (18.2%)		262 (16.2%)	250 (15.4%)	
**HER2 status**												
Negative	570 (43.4%)	552 (42.0%)	0.052	762 (58.0%)	360 (27.4%)	<**0.001**	636 (41.2%)	779 (50.5%)	**0.025**	750 (48.6%)	665 (43.1%)	0.113
Positive	83 (6.3%)	109 (8.3%)		104 (7.9%)	88 (6.7%)		44 (2.9%)	83 (5.4%)		58 (3.8%)	69 (4.5%)	
**Triple negative disease**												
Negative	558 (42.6%)	515 (39.3%)	**0.001**	734 (56.0%)	339 (25.9%)	<**0.001**	640 (39.7%)	733 (45.5%)	<**0.001**	753 (46.7%)	620 (38.5%)	<**0.001**
Positive	96 (7.3%)	142 (10.8%)		130 (9.9%)	108 (8.2%)		67 (4.2%)	171 (10.6%)		85 (5.3%)	153 (9.5%)	
**Vascular invasion**												
Negative	430 (32.2%)	464 (34.7%)	0.141	582 (43.5%)	312 (23.3%)	0.589	481 (29.4%)	678 (41.4)	**0.007**	568 (34.7%)	591 (36.1%)	<**0.001**
Positive	232 (17.4%)	211 (15.8%)		295 (22.1%)	148 (11.1%)		233 (14.2%)	245 (15.0%)		278 (17.0%)	200 (12.2%)	

The *P* values are resultant from Pearson χ^2^ test of association and significant values (*P* < 0.05) are highlighted in bold. ER is oestrogen receptor and PgR is progesterone receptor.

### Validation cohort

1655 early stage invasive breast cancer patients were available for assessment in the validation cohort, with all patients treated at Nottingham University Hospitals between 1998 and 2006. All patients were managed in a standard manner, as described for the discovery cohort. Median follow-up was 148 months determined using the reverse Kaplan-Meier method and clinicopathological information for this cohort is available in Table 1.

### HER2 positive cohort

112 HER2 positive breast cancer patients were available for assessment in the HER2 positive cohort, with all patients treated at Nottingham University Hospitals between 2004 and 2012. Patients were treated according to local guidelines, with adjuvant therapy and trastuzumab following surgery. Adjuvant hormone therapy was received by 47% of patients (40/75), with 74% of patients receiving adjuvant radiotherapy (59/80). Trastuzumab was given on a 3-weekly regimen for 52 weeks, with patients receiving trastuzumab following six cycles of 3-weekly FEC chemotherapy (fluorouracil, epirubicin and cyclophosphamide) or patients receiving three cycles of FEC, followed by three cycles of taxane (FEC-T), to which, trastuzumab was frequently added from the second cycle of taxane onwards. Median follow-up was 50 months determined using the reverse Kaplan-Meier method and clinicopathological information for this cohort is available in Table 2.

**Table 2:** Associations between the expression of DARPP-32 determined in HER2 positive breast cancer patients treated with trastuzumab and adjuvant chemotherapy and clinicopathological variables.

		Cytoplasmic			Nuclear		
		Low	High	*P* value	Low	High	*P* value
Age	≤40 years	6 (5.4%)	9 (8.0%)	0.710	6 (5.4%)	9 (8.0%)	0.651
	>40 years	34 (30.4%)	63 (56.3%)		33 (29.5%)	64 (57.1%)	
Tumour size	≤20 mm	24 (21.6%)	44 (39.6%)	0.965	24 (21.6%)	44 (39.6%)	0.767
	>20 mm	15 (13.5%)	28 (25.2%)		14 (12.6%)	29 (26.1%)	
Node status	Negative	16 (14.3%)	28 (25.0%)	0.908	15 (13.4%)	29 (25.9%)	0.896
	Positive	24 (21.4%)	44 (39.3%)		24 (21.4%)	44 (39.3%)	
T stage	0	0 (0.0%)	1 (0.9%)	0.644	0 (0.0%)	1 (0.9%)	0.684
	1	25 (22.3%)	44 (39.3%)		25 (22.3%)	44 (39.3%)	
	2	14 (12.5%)	22 (19.6%)		13 (11.6%)	23 (20.5%)	
	3	1 (0.9%)	5 (4.5%)		1 (0.9%)	5 (4.5%)	
Tumour grade	1	0 (0.0%)	3 (2.7%)	0.424	0 (0.0%)	3 (2.7%)	0.430
	2	13 (11.6%)	22 (19.6%)		13 (11.6%)	22 (19.6%)	
	3	27 (24.1%)	47 (42.0%)		26 (23.2%)	48 (42.9%)	
ER status	Negative	14 (12.5%)	36 (32.1%)	0.126	14 (12.5%)	36 (32.1%)	0.174
	Positive	26 (23.2%)	36 (32.1%)		25 (22.3%)	37 (33.0%)	
PgR status	Negative	16 (17.2%)	43 (46.2%)	0.163	16 (17.2%)	43 (46.2%)	0.265
	Positive	14 (15.1%)	20 (21.5%)		13 (14.0%)	21 (22.6%)	
NPI category	Good (≤3.4)	1 (1.0%)	6 (5.9%)	0.417	2 (2.6%)	5 (5.0%)	0.385
	Intermediate (3.41–5.4)	27 (26.7%)	42 (41.6%)		23 (22.8%)	46 (45.5%)	
	Poor (>5.4)	10 (9.9%)	15 (14.9%)		12 (11.9%)	13 (12.9%)	

The *P* values are resultant from Pearson χ^2^ test of association and significant values (*P* < 0.05) are highlighted in bold. ER is oestrogen receptor and PgR is progesterone receptor.

### METABRIC series

Details of the Molecular Taxonomy of Breast Cancer International Consortium (METABRIC) data set (n = 1980) data set have been published elsewhere^27^. Tumours were collected by five centres in the UK and Canada between 1977–2005 and almost all ER negative and lymph node positive patients received adjuvant chemotherapy, whereas ER negative and/or lymph node positive patient did not. No patients with HER2 overexpression received trastuzumab. Median follow-up was 141 months determined using the reverse Kaplan-Meier method. DNA and RNA were isolated from samples and hybridised to the Affymetrix SNP 6.0 and Illumina HT-12 v3 platforms for genomic and transcriptional profiling as described by Curtis *et al*. (2012)^27^. This cohort was used to assess the prognostic significance of DARPP-32 at the mRNA level and determine associations with other genes using artificial neural network analysis.

### Immunohistochemistry

Immunohistochemistry was performed on tissue microarrays for the discovery cohort, validation cohort and the HER2 cohort, and were comprised of single 0.6 mm cores taken from a representative tumour area as assessed on Haematoxylin and Eosin stained sections by a specialist breast cancer histopathologist. Immunohostochemical staining was achieved using a Novolink Polymer Detection kit (Leica) according to the manufacturers’ instructions, the use of which has been described previously^28–30^. In brief, xylene was used to deparaffinise tissue, followed by rehydration in ethanol then water. Antigen retrieval was achieved in 0.01molL^−1^ sodium citrate buffer (pH6.0), heated in a microwave for 10 minutes at 750 W followed by 10 minutes at 450 W. Tissue was treated with Novolink Peroxidase Block, washed with Tris-buffered saline (TBS), and then treated with Novolink Protein Block solution. Rabbit polyclonal anti-DARPP-32 (Abcam ab40801) diluted 1:500 was used as primary antibody and was incubated on tissue for one hour at room temperature. Antibody specificity was confirmed by Western blotting on breast cancer cell lysates prior to use. Tissue was washed with TBS prior to the application of Novolink Post Primary solution, which was subsequently washed with TBS and then Novolink Polymer solution was applied. Immunohistochemical reactions were developed using 3,3′ diaminobenzidine as the chromogenic substrate and tissue was counterstained with haematoxylin. Tissue was dehydrated in ethanol and fixed in xylene prior to mounting using DPX. Positive and negative controls were included with each staining run and were comprised of breast tumour composite sections comprising grade 1 and 2 early stage invasive tumour; negative controls had primary antibody omitted from each staining run (Supplementary Information).

### Statistical analyses

Slides were scanned using a Nanozoomer Digital Pathology Scanner (Hamamatsu Photonics) and staining was assessed at 200x magnification. Staining in the cytoplasm was assessed using a semi-quantitative immunohistochemical H score, where staining intensity within tumour cells was assessed as none (0), weak (1), medium (2) or strong (3) over the percentage area of each staining intensity. Staining in the nucleus was examined in a semi-quantitative manner, where the percentage of tumour cells that demonstrated any staining intensity was assessed. Greater than 30% of cores for each TMA were double assessed, with both assessors blinded to clinical outcome and each other’s scores. Single measure intraclass correlation coefficients were above 0.7, indicating good concordance between scorers.

Statistical analysis was performed using IBM SPSS Statistics (version 24). Cases were stratified based on breast cancer specific survival for both the discovery and validation cohorts and the METABRIC cohort, and relapse-free survival for the HER2 positive cohort, using X-Tile software^31^. All differences were deemed statistically significant at the level of *P* ≤ 0.05. The Pearson χ^2^ test of association was used to determine the relationship between categorised protein expression and clinicopathological variables. Survival curves were plotted according to the Kaplan-Meier method with significance determined using the log-rank test.

### Identification of genes associated with DARPP-32 expression

A supervised artificial neural network was used to further understand the molecular function of *PPP1R1B* in breast cancer in the METABRIC series. *PPP1B1R* expression was used as the supervising variable as described by Abdel-Fatah *et al*.^32^. The artificial neural network was conducted with a constrained multi-layer perceptron architecture and sigmoidal transfer function, where weights were updated by a feed forward back propagation algorithm. Probes from the METABRIC data were ranked based on their root mean squared (RMS) error for predication of DARPP-32 expression as a continuous variable.

## Results

### DARPP-32 protein staining location and frequency

**Figure 1:**
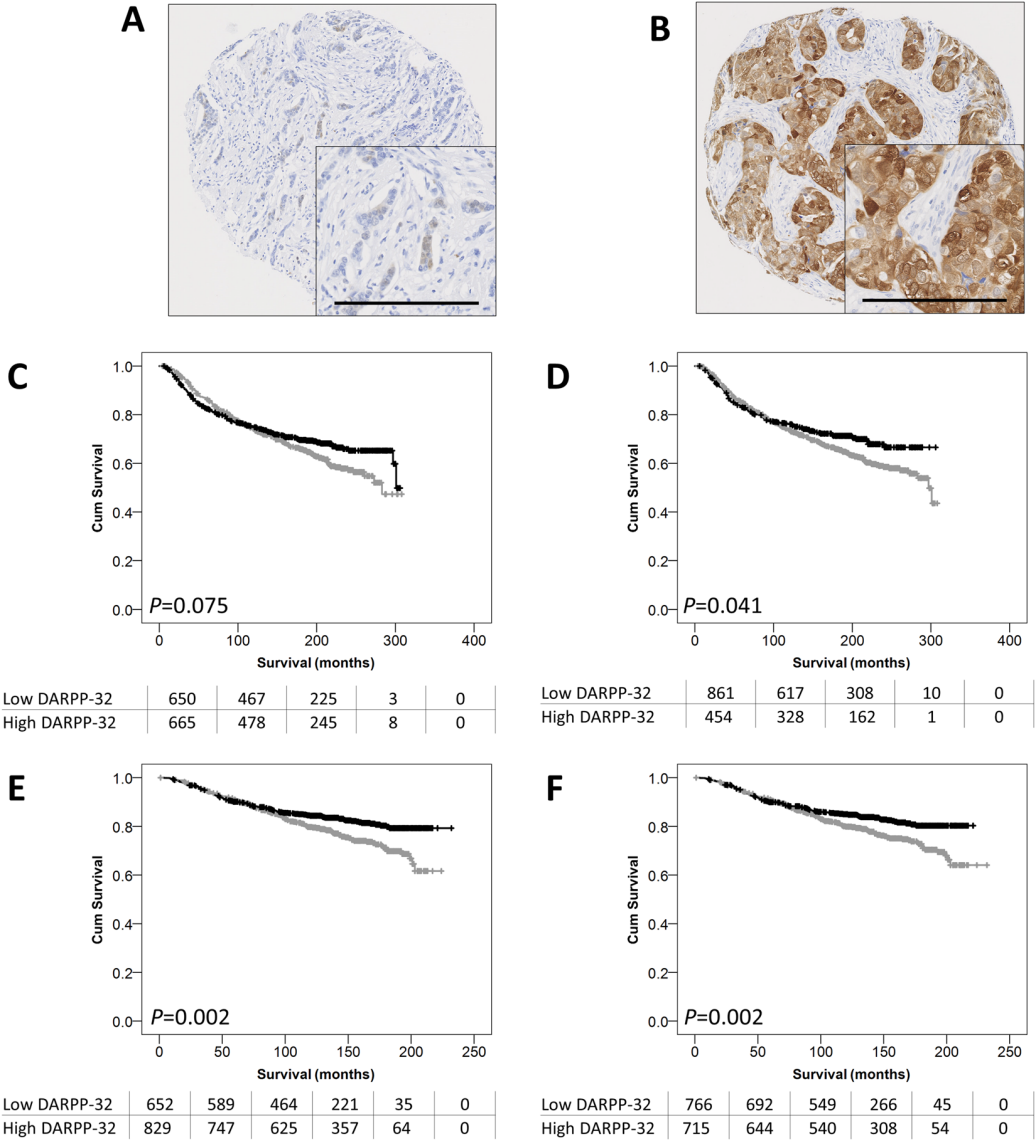
Representative photomicrographs DARPP-32 staining. Photomicrographs of low DARPP-32 immunohistochemical staining (**A**), and high staining (**B**) are shown at 100x magnification with 200x magnification inset box where the scale bar represents 100 µm. Kaplan-Meier analysis of breast cancer specific survival showing the impact of low (grey line) and high (black line) DARPP-32 protein expression within the cytoplasm (**C**) or the nucleus (**D**) in the discovery cohort, and within the cytoplasm (**E**) or the nucleus (**F**) in the validation cohort. Significance was determined using the log-rank test. The numbers shown below the Kaplan-Meier survival curves are the number of patients at risk at the specified month.

DARPP-32 protein expression was assessed in two large independent cohorts of early invasive breast cancer. Nuclear and cytoplasmic DARPP-32 expression was observed in all cohorts and varied from weak to intense, with heterogeneity observed between adjacent tumour cells. Representative photomicrographs are shown in Fig. 1. In the discovery cohort, 1352 patients were assessed, cytoplasmic DARPP-32 expression had a median H-score of 40, and ranged from 0 to 300. In the validation cohort, 1655 patients were assessed, cytoplasmic DARPP-32 expression had a median H score of 20 and ranged from 0 to 300. Nuclear DARPP-32 expression in the discovery cohort had a median H score of 0 and ranged from 0 to 100; in the validation cohort, the median DARPP-32 H-score was 5 and ranged from 0 to 100. X-tile was used to generate cut points for use in both cohorts based on breast cancer specific survival. In the discovery cohort, cytoplasmic DARPP-32 expression had a cut point of 35, with 49.6% of cases (670/1352) demonstrating low expression; in the validation cohort a cut point of 10 was used, with 43.6% of cases (722/1655) demonstrating low expression. In the discovery cohort, nuclear DARPP-32 expression had a cut point of 20, with 65.6% of cases (887/1352) demonstrating low expression; in the validation cohort, a cut point of 10 was used, with 51.5% of cases (853/1655) demonstrating low expression.

### Relationship between DARPP-32 protein expression and clinicopathological variables

In the discovery cohort, low cytoplasmic DARPP-32 expression was significantly associated with ER and PgR positive tumours (χ^2^ = 25.893, d.f. = 1, *P* < 0.001 and χ^2^ = 7.384, d.f. = 1, *P* = 0.007 respectively) and absence of triple negative disease (χ^2^ = 10.607, d.f. = 1, *P* = 0.001) (Table 1). In the validation cohort, low cytoplasmic DARPP-32 expression was significantly associated with larger tumour size (χ^2^ = 3.917, d.f. = 1, *P* = 0.048), higher tumour grade (χ^2^ = 17.517, d.f. = 2, *P* < 0.001), tubule formation (χ^2^ = 34.097, d.f. = 2, *P* < 0.001), pleomorphism (χ^2^ = 3.917, d.f. = 2, *P* = 0.016), lower mitosis (χ^2^ = 6.785, d.f. = 2, *P* = 0.034), increased tumour stage (χ^2^ = 8.215, d.f. = 2, *P* = 0.016), ER and PgR positive tumours (χ^2^ = 39.000, d.f. = 1, *P* < 0.001 and χ^2^ = 13.987, d.f. = 1, *P* < 0.001 respectively), belonging to a poor NPI prognostic group (χ^2^ = 12.386, d.f. = 2, *P* = 0.002) and HER2 status (χ^2^ = 5.017, d.f. = 1, *P* = 0.025), absence of triple negative disease (χ^2^ = 28.075, d.f. = 1, *P* < 0.001), and the presence of lymphovascular invasion (χ^2^ = 7.220, d.f. = 1, *P* = 0.007) (Table 1).

In the discovery cohort, low nuclear DARPP-32 expression was significantly associated with increased pleomorphism (χ^2^ = 6.943, d.f. = 2, *P* = 0.031), ER and PgR positive tumours (χ^2^ = 51.128, d.f. = 1, *P* < 0.001 and χ^2^ = 22.736, d.f. = 1, *P* < 0.001 respectively), HER2 negative tumours (χ^2^ = 13.790, d.f. = 1, *P* < 0.001), and the absence of triple negative disease (χ^2^ = 16.472, d.f. = 1, *P* < 0.001) (Table 1). In the validation cohort, low nuclear DARPP-32 expression was significantly associated with higher grade tumours (χ^2^ = 17.859, d.f. = 2, *P* < 0.001), tubule formation (χ^2^ = 26.145, d.f. = 2, *P* < 0.001), lower mitosis (χ^2^ = 10.070, d.f. = 2, *P* = 0.007), higher tumour stage (χ^2^ = 14.358, d.f. = 2, *P* = 0.001), ER and PgR positive tumours (χ^2^ = 41.180, d.f. = 1, *P* < 0.001 and χ^2^ = 15.031, d.f. = 1, *P* < 0.001 respectively), belonging to a poor NPI prognostic group (χ^2^ = 21.111, d.f. = 2, *P* < 0.001), the absence of triple negative disease (χ^2^ = 28.738, d.f. = 1, *P* < 0.001), and the presence of lymphovascular invasion (χ^2^ = 11.349, d.f. = 1, *P* = 0.001) (Table 1).

### Association between DARPP-32 protein expression and survival

In the discovery cohort, low nuclear expression of DARPP-32 was significantly associated with adverse breast cancer specific survival (*P* = 0.041) (Fig. 1D). Low nuclear expression of DARPP-32 remained significantly associated with adverse survival (hazard ratio (HR): 0.766; 95% confidence interval (CI): 0.613–0.957; *P* = 0.019) when potentially confounding factors were included in multivariate assessment (including tumour size, stage, grade, NPI status, vascular invasion status, ER, PgR and HER2 receptor status (all with log-rank statistics of *P* < 0.001) (Table 2).

In the validation cohort, both low cytoplasmic and low nuclear DARPP-32 expression were significantly associated with survival (both *P* = 0.002) (Fig. 1E,F). Cytoplasmic expression of DARPP-32 remained significantly associated with adverse survival (HR: 0.744; 95% CI: 0.577–0.960; *P* = 0.023) when the potentially confounding factors were included in multivariate assessment (including tumour size, stage, grade, NPI status, vascular invasion status ER, PgR and HER2 receptor status (all with log rank statistics of *P* = 0.001). Nuclear expression of DARPP-32 did not remain significantly associated with survival in the validation cohort (HR: 0.786; 95% CI: 0.608–1.016; *P* = 0.066).

### DARPP-32 expression in ER positive disease

**Figure 2:**
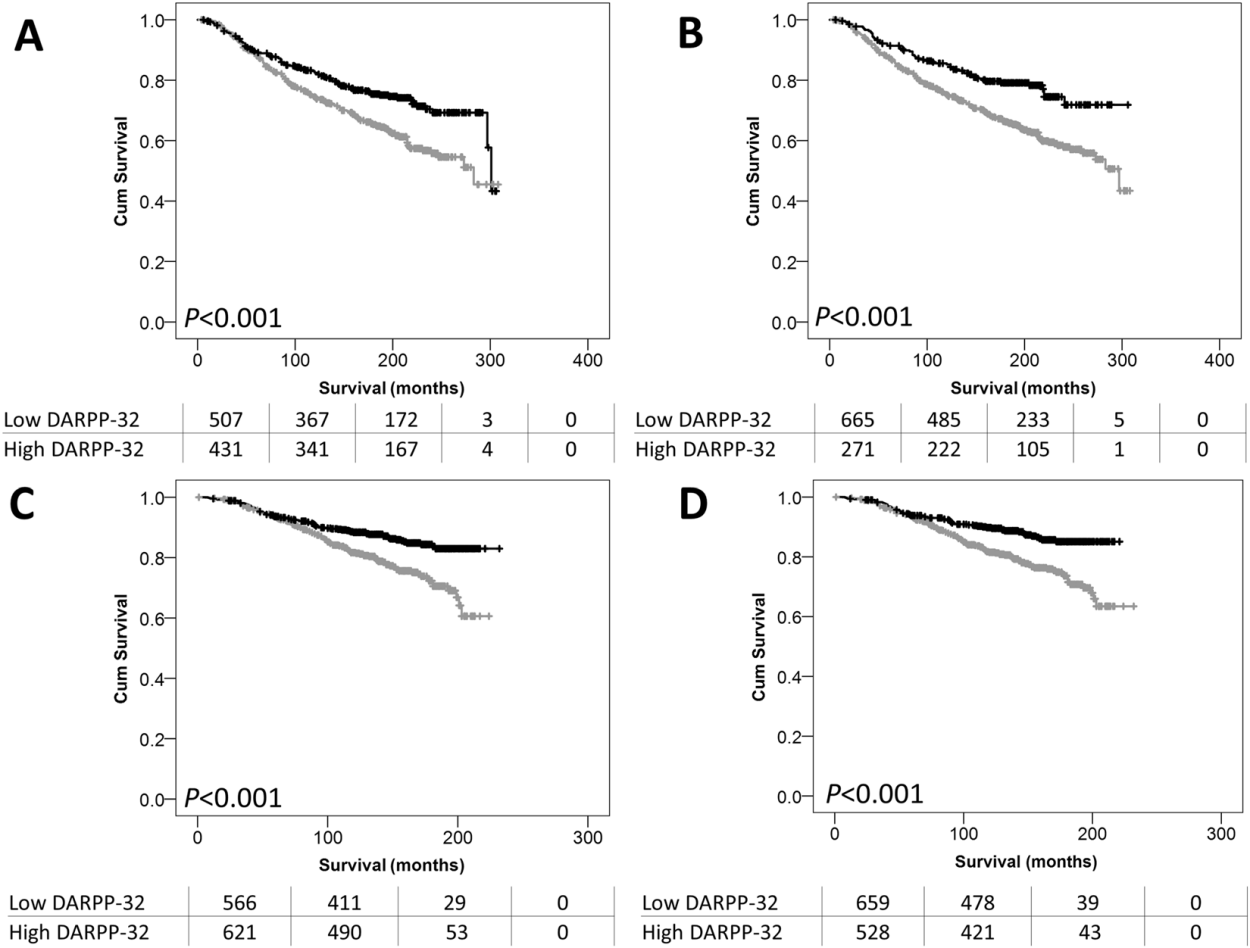
DARPP-32 expression association with patient outcome. Kaplan-Meier analysis of breast cancer specific survival showing the impact of low (grey line) and high (black line) DARPP-32 expression within the cytoplasm in ER positive patients (**A**) and in the nucleus in ER positive patients (**B**) in the discovery cohort. DARPP-32 expression within the cytoplasm in ER positive patients (**C**) and in the nucleus in ER positive patients (**D**) in the validation cohort. Significance was determined using the log-rank test. The numbers shown below the Kaplan-Meier survival curves are the number of patients at risk at the specified month.

Low expression of DARPP-32 was particularly important in patients with ER positive disease. In the discovery cohort, low DARPP-32 cytoplasmic expression was significantly associated with adverse survival of ER positive patients (*P* < 0.001), but not ER negative patients (*P* = 0.099) (Fig. 2A). The same finding was observed when nuclear DARPP-32 expression was assessed, with low expression significantly associated with adverse survival of ER positive patients (*P* < 0.001), but not ER negative patients (*P* = 0.407) (Fig. 2B). In the validation cohort, low cytoplasmic expression of DARPP-32 associated with adverse survival of ER positive patients (*P* < 0.001), but not ER negative patients (*P* = 0.291) (Fig. 2C). Similar findings were for nuclear DARPP-32 expression were observed; with low expression associated with adverse survival of ER positive patients (*P* < 0.001), but not ER negative patients (*P* = 0.679) (Fig. 2D).

### DARPP-32 expression in HER2 positive patients treated with trastuzumab

DARPP-32 was assessed in a cohort of 112 HER2 positive breast cancer patients treated with adjuvant chemotherapy and trastuzumab. A similar DARPP-32 expression pattern was observed to that in early breast cancer. In this cohort, cytoplasmic DARPP-32 had a median H score of 142.9 and ranged from 0 to 300; nuclear DARPP-32 expression had a median H score of 45.36 and ranged from 0 to 100. X-tile was used to generate a cut point for analysis based on relapse free survival; cytoplasmic DARPP-32 expression had a cut point of 20, with 35.7% of cases (40/112) demonstrating low expression, nuclear DARPP-32 expression had a cut point of 5, with 34.8% of cases (39/112) demonstrating low expression.

**Figure 3:**
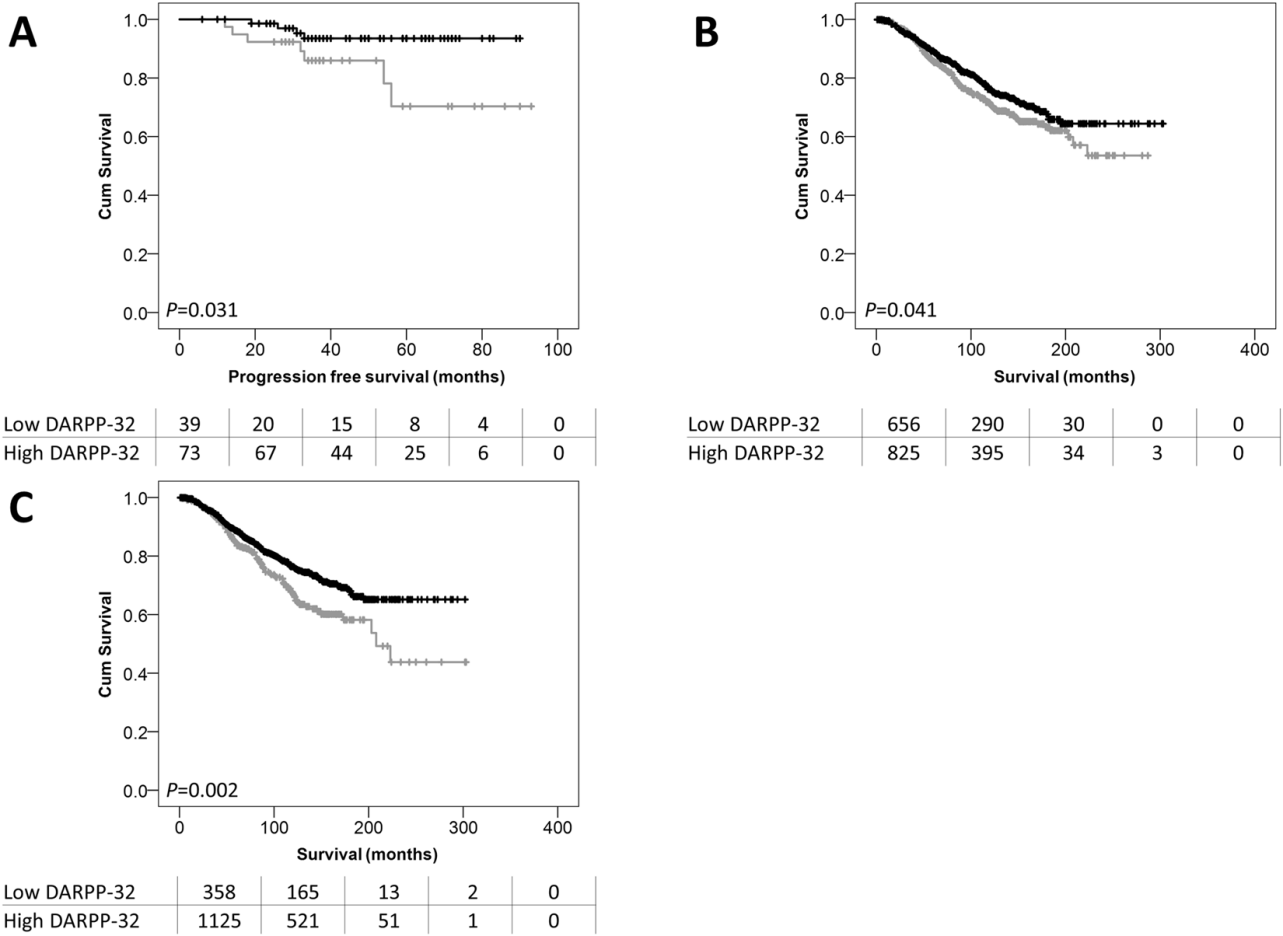
DARPP-32 expression association with HER2 positive patient outcome. Kaplan-Meier analysis of progression-free survival of HER2 positive breast cancer patients treated with adjuvant chemotherapy and trastuzumab showing the impact of high (black line) and low (grey line) DARPP-32 protein expression within the nucleus (**A**). Kaplan-Meier analysis of PPP1R1B probe 2 (**B**) and probe 3 (**C**) expression in ER positive breast cancer patients showing the impact of low expression (grey line) and high expression (black line) and breast cancer specific survival. Significance was determined using the log-rank test. The numbers shown below the Kaplan-Meier survival curves are the number of patients at risk at the specified month.

No associations were observed between DARPP-32 expression and clinicopathological criteria in this cohort (Table 2). Low nuclear DARPP-32 expression was significantly associated with adverse progression-free survival of HER2 positive breast cancer patients treated with trastuzumab (*P* = 0.031) (Fig. 3A); cytoplasmic DARPP-32 expression was not associated with progression-free survival (data not shown). Nuclear DARPP-32 expression did not remain associated with progression-free survival in multivariate Cox regression (HR = 0.387; 95% CI = 0.095–1.570; *P* = 0.184); when tumour size, lymph node status and NPI category were included (with individual Kaplan-Meier log rank significance of *P* = 0.012, *P* = 0.019 and *P* = 0.008 respectively).

### PPP1R1B mRNA expression and patient survival

Three *PPP1R1B* probes were available to assess mRNA expression in the METABRIC cohort and expression was categorised using the median gene expression value to assess for association with patient survival and to perform expression profiling. *PPP1R1B* probe 1 (ILMN_1690096) is located within a coding area that corresponds to the N-terminal region of DARPP-32; *PPP1R1B* probe 2 (ILMN_1759012) and *PPP1R1B* probe 3 (ILMN_2304495) were both located in untranslated regions (5′ and 3′ respectively). Probe 1 and 3 are located in areas found in the sequence for DARPP-32 (NM_032192), and probe 2 and 3 are located in areas found in the sequence for t-DARPP (NM_181505.3). No association was observed between the expression of the probes and disease specific survival. The gene expression data was analysed using an artificial neural network approach that uses a machine learning based data mining algorithm. A rank order of all the genes was produced based on the minimum average root mean squared error. The top 200 transcripts were selected for *PPP1R1B* probe 1, 2 and 3 and 73 common transcripts were identified (Table 3). The transcripts that were common to all three probes included *CDC42*, *DKK1*, *GRB7*, *PNMT*, and *GPER* amongst others.

**Table 3:** Genes associated with two or more *PPP1R1B* probes in the METABRIC dataset identified by artificial neural network analysis. Those highlighted in bold were associated with expression of all three *PPP1R1B* probes.

Gene	Illumina ID	Identity	Gene	Illumina ID	Identity
AFF3	ILMN_1775235	AF4/FMR2 family member 3	NEDD9	ILMN_1726164	Cas scaffolding protein family member 2
AMDHD1	ILMN_1788239	Amidohydrolase domain containing 1	OAT	ILMN_1654441	Ornithine aminotransferase
**ATP13A5**	**ILMN_1775285**	ATPase 13A5	**OAT**	**ILMN_2068747**	
BBOX 1	ILMN_1734929	Gamma-butyrobetaine hydrolase 1	**OBP2B**	**ILMN_1700666**	Odorant binding protein 2B
C22orf36	ILMN_1737255	Leucine rich repeat containing 75B	ORM2	ILMN_1731785	Orosomucoid 2
CDC42	ILMN_1696041	Cell division cycle 42	PAQR6	ILMN_1689852	Progestin and adipoQ receptor family member 6
CEACAM1	ILMN_1716815	Carcinoembryonic antigen related cell adhesion molecule 1	PDE4B	ILMN_1782922	Phosphodiesterase 4B
CITED4	ILMN_1787691	Cbp/p300 interacting transactivator 4	**PNMT**	**ILMN_1710027**	Phenylethanolamine N-methyltransferase
CPA4	ILMN_1784294	Carboxypeptidase A4	**PPP1R1A**	**ILMN_2056606**	Protein phosphatase 1 regulatory inhibitor subunit 1A
**CRABP1**	**ILMN_1658040**	Cellular reinoic acid binding protein 1	**PROM1**	**ILMN_1786720**	Prominin1
CYP4Z1	ILMN_1693594	Cytochrome P450 family 4 subfamily Z member 1	PVRL4	ILMN_1749044	Nectin cell adhesion molecule 4
CYP4Z1	ILMN_1728550		RGMA	ILMN_1717636	Repulsive guidance molecule BMP co-receptor A
CYP4Z1	ILMN_2359698		**RNF183**	**ILMN_1692591**	Ring finger protein 183
CYP4Z2P	ILMN_1702829	Putative inactive cytochrome P450 family member 4Z2	S100A1	ILMN_1653494	S100 calcium binding protein A1
DCD	ILMN_1722554	Dercidin	S100A13	ILMN_1738707	S100 calcium binding protein A13
DKK1	ILMN_1773337	Dickkopf WNT signalling pathway inhibitor 1	SASH1	ILMN_1712673	SAM and SH3 domin containing 1
ENPP3	ILMN_1749131	Ectonucleotide pyrophosphatase/phosphodiesterase 3	SASH1	ILMN_2185984	
FAIM2	ILMN_1803855	Fas apoptotic inhibitory molecule 2	SCGB2A2	ILMN_1723333	Secretoglobulin family 2 A member 2
**FAM134B**	**ILMN_1811330**	Reticulophagy regulator 1	SLC22A15	ILMN_1730639	Solute carrier family 22 member 15
FAM134B	ILMN_2283597		SLC25A18	ILMN_1754864	Solute carrier family 25 member 18
FAM134B	ILMN_2387952		SLC5A1	ILMN_1681526	Solute carrier family 5 member 1
FAT	ILMN_1754795	FAT atypical cadherin 1	**SOX9**	**ILMN_1805466**	SRY box 9
FOLR1	ILMN_2346339	Folate receptor 1	SPINK8	ILMN_1728898	Serine peptidase inhibitor, Kazal type 8
**GGT6**	**ILMN_1788942**	Gamma-glutamyltransferase 6	SPRY1	ILMN_1691860	Sprouty RTK signaling antagonist 1
**GPER**	**ILMN_1795298**	G protein-coupled oestrogen receptor 1	ST6GAL1	ILMN_1756501	ST6 beta-galactosidase alpha-2,6-sialytransferase 1
GRAMD2	ILMN_1661443	GRAM domain containing 2A	**STAC2**	**ILMN_1718295**	SH3 and cysteine rich domain 2
GRB7	ILMN_1740762	Growth factor receptor bound protein 7	**TFAP2B**	**ILMN_1758404**	Transcription factor AP-2 beta
HOXA5	ILMN_1753613	Homeobox A5	TFAP2B	ILMN_1853592	
**HSD17B2**	**ILMN_1808713**	Hydroxysteroid 17 beta dehydrogenase 2	TRPV6	ILMN_1674533	Epithelial calcium channel 2
**HSH2D**	**ILMN_1788017**	Hematopoietic SH2 domain containing	**TSPAN6**	**ILMN_1730998**	Tetraspannin 6
ICAM1	ILMN_1812226	Intracellular adhesion molecule 1	TTLL4	ILMN_1746846	Tubulin tyrosine ligase like 4
IGSF9	ILMN_1693941	Immunoglobulin superfamily member 9	UBE2E3	ILMN_1669553	Ubiquitin conjugating enzyme E2 E3
KRT7	ILMN_2163723	Keratin 7	UBE2E3	ILMN_2390338	
LOC340204	ILMN_1789600		VTCN1	ILMN_1753101	V-set containing T cell activation inhibitor 1
**LOC646424**	**ILMN_1661466**			ILMN_1854349_	
LOC730525	ILMN_1651610			ILMN_1889752_	
MAOB	ILMN_1727360	Monoamine oxidase B		ILMN_1896906_	
MAST4	ILMN_1738438	Microtubule associated Ser/Thr kinase family member 4		ILMN_1902123_	
MPZL2	ILMN_1752932	Myelin protein zero like 2		ILMN_1904054_	

All three *PPP1R1B* probes were further assessed in ER positive patients based on the observed DARPP-32 protein findings, with a cut point generated in this subgroup of patients using X-tile. *PPP1R1B* probe 1 expression was not associated with breast cancer specific survival of ER positive patients; however, low expression of *PPP1R1B* probe 2 and 3 were both associated with adverse survival of ER positive breast cancer patients (*P* = 0.041 and *P* = 0.002 respectively) (Fig. 3B,C).

## Discussion

Low nuclear DARPP-32 expression was significantly associated with adverse survival of patients in two independent cohorts of patients treated at Nottingham University Hospitals (discovery cohort n = 1352 and validation cohort n = 1655). Furthermore, low cytoplasmic expression of DARPP-32 was associated with patient survival in the validation cohort. The epitope for the antibody used for immunohistochemistry is located within amino acids 0–30, meaning that only DARPP-32, and not t-DARPP expression was assessed in this study. There are limited reports of DARPP-32 expression and its association with patient survival in cancer. In oesophageal squamous cell carcinoma DARPP-32 is expressed after a phase of dysplasia, and low levels of DARPP-32 are associated with tumours that progress rapidly^33^. In colorectal cancer, lower expression of DARPP-32 is associated with improved overall survival and disease free survival^12^, and in non-small cell lung cancer high relative t-DARPP (in comparison to DARPP-32) is associated with tumour stage^34^. The expression of DARPP-32 in 230 breast cancer patients has been examined previously using N-terminal and C-terminal specific antibodies, to show that high N-terminal DARPP-32 expression is associated with adverse patient survival; this differs from the results presented here^21^. It is unclear why opposing results were observed; however, patient demographics, including clinicopathological variables, and patient treatment are not available in the previously published study, so cannot be directly compared with the current findings.

Interestingly, low DARPP-32 protein expression was particularly important in ER positive patients, in both cohorts of patients; there was also a strong association between DARPP-32 expression and ER receptor status. In addition to ER status, strong consistent associations were observed between DARPP-32 expression and PgR status and triple receptor negative disease in both patient cohorts.

There is accumulating evidence that DARPP-32, in particular t-DARPP, plays a role in response to trastuzumab; this study shows that low nuclear expression of DARPP-32 is significantly associated with adverse progression free survival in 112 HER2 positive patients treated with trastuzumab and adjuvant chemotherapy. This is agreement with published *in vitro* data, that indicates expression of DARPP-32 and t-DARPP expression in HER2 positive breast cancer is involved in resistance to trastuzumab^22^. Expression of t-DARPP has been shown to activate IGF-1R signalling in trastuzumab resistant breast cancer cells through increased glycolytic capacity^35^. In addition, DARPP-32 mRNA and protein levels have also been shown to fall in HER2 targeted agent, lapatinib, resistant breast cancer cell lines, with a t-DARPP mediated survival advantage observed^24^.

In models of murine tumourigenesis DARPP-32 expression is expressed in normal mouse tissue and some breast tumours, with t-DARPP expressed only within tumours^18^. It is interesting to hypothesise that the association between patient survival and low expression of DARPP-32 is observed with a corresponding shift to increased t-DARPP expression; this will be determined in future studies.

PPP1R1B expression was assessed in the METABRIC cohort, where three probes were available for assessment, probe 1 and 3 located in DARPP-32 and probe 2 and 3 located in t-DARPP. Artificial neural network analysis identified a number of genes associated with *PPP1R1B* expression. Artificial neural network analysis was performed using all three *PPP1R1B* probes and commonalities within the top 200 genes for each probe identified. Validation of these associations will be performed as part of future studies. Interestingly, *CDC42* was identified from this analysis; cdc42 plays a role in filopodia formation and breast cancer cells expressing DARPP-32 have, in a study looking at Wnt-5A activation of DARPP-32, been shown to have lower cdc-42 activity^17^. In addition to *CDC42*, Dickkopf-1 (*DKK1*) was also identified and functions a wnt-5A pathway inhibitor. Furthermore, artificial neural network analysis identified an association between *PPP1R1B* with both *GRB7* and *PNMT*, a link between *PPP1R1B* and these genes has already been described in upper gastrointestinal adenocarcinomas where DNA amplification at 17q is often detected (containing *PPP1R1B*, *STARD3*, *TCAP*, *PNMT*, *PERLD1*, *ERBB2*, *C17orf37*, and *GRB7*)^36^.

## Conclusion

This study demonstrates that low DARPP-32 protein expression is associated with shorter survival in two large, independent, early stage invasive breast cancer patient cohorts, with a stronger association observed in ER positive disease. This finding was also observed at the mRNA level, with low *PPP1R1B* expression significantly associated with shorter survival of ER positive patients in the METABRIC cohort. Furthermore, low DARPP-32 expression was associated with shorter progression-free survival of HER2 positive patients treated with trastuzumab. This data suggests a potential role for DARPP-32 as a prognostic marker with clinical utility in breast cancer, requiring validation on samples from multiple institutions.

### Compliance with ethical standards

#### Research involving human participants

Ethical approval for the discovery cohort, validation cohort and the HER 2 cohort was granted by Nottingham Research Ethics Committee 2, under the title ‘Development of a molecular genetic classification of breast cancer’ (C202313). METABRIC samples were collected by five centres in the UK and Canada and were acquired with appropriate consent from the respective institutional review boards^27^. All procedures performed in studies involving human participants were in accordance with the ethical standards of the institutional and/or national research committee and with the 1964 Helsinki declaration and its later amendments or comparable ethical standards. All samples collected from Nottingham used in this study were pseudo-anonymised; those collected prior to 2006 did not require informed patient consent under the Human Tissue Act, after 2006 informed consent was obtained from all individual participants included in the study.

## Supplementary information

Supplementary Figure 1

## Data Availability

The METABRIC data is publically available https://www.ebi.ac.uk/ega/studies/EGAS00000000098. Immunohistochemistry datasets analysed during the current study are available from the corresponding author on reasonable request.
