# Supplementary material for: Dopamine and cAMP-regulated phosphoprotein 32 kDa (DARPP-32) and survival in breast cancer: a retrospective analysis of protein and mRNA expression
Source: Sci Rep. 2019 Nov 18;9:16987. doi: 10.1038/s41598-019-53529-z (PMC6861271; doi:10.1038/s41598-019-53529-z)
Supplement: Supplementary file 1 — Supplementary Figure 1 [file 41598_2019_53529_MOESM1_ESM.docx]

**Dopamine and cAMP-regulated phosphoprotein 32kDa (DARPP-32) and survival in breast cancer: a retrospective analysis of protein and mRNA expression**

Shreeya Kotecha, Marie N Lebot, Bhudsaban Sukkarn, Graham Ball, Paul M Moseley, Stephen Y Chan, Andrew R Green, Emad Rakha, Ian O Ellis, Stewart G Martin, Sarah J Storr


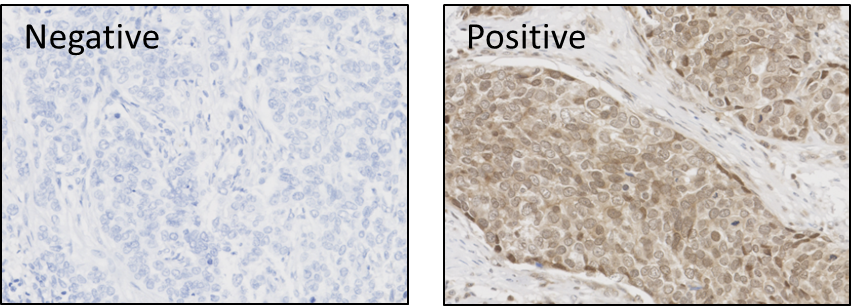


Supplementary Figure 1:

Representative photomicrographs of negative and positive control staining in breast cancer tissue (magnification at 100x).
